# Correction: Advances in metal–organic frameworks for water remediation applications

**DOI:** 10.1039/d4ra90018a

**Published:** 2024-03-05

**Authors:** Seema Lal, Parul Singh, Anchal Singhal, Sanjay Kumar, Ajay Pratap Singh Gahlot, Namita Gandhi, Pratibha Kumari

**Affiliations:** a Department of Chemistry, Deshbandhu College, University of Delhi New Delhi India; b Department of Chemistry, St. Joseph’s University Bengaluru Karnataka India; c Department of Physics, Deshbandhu College, University of Delhi New Delhi India pkumari@db.du.ac.in

## Abstract

Correction for ‘Advances in metal–organic frameworks for water remediation applications’ by Seema Lal *et al.*, *RSC Adv.*, 2024, **14**, 3413–3446, https://doi.org/10.1039/D3RA07982A.

The authors regret that one of the affiliations (affiliation *b*) was incorrectly shown in the original manuscript. The corrected list of affiliations is as shown here.

The Royal Society of Chemistry apologises for these errors and any consequent inconvenience to authors and readers.

## Supplementary Material

